# A Unique Carrier for Delivery of Therapeutic Compounds beyond the Blood-Brain Barrier

**DOI:** 10.1371/journal.pone.0002469

**Published:** 2008-06-25

**Authors:** Delara Karkan, Cheryl Pfeifer, Timothy Z. Vitalis, Gavin Arthur, Maki Ujiie, Qingqi Chen, Sam Tsai, Gerrasimo Koliatis, Reinhard Gabathuler, Wilfred A. Jefferies

**Affiliations:** 1 BioMarin Pharmaceutical Inc., Vancouver, Canada; 2 Department of Medical Genetics, the Michael Smith Laboratories and the Biomedical Research Centre, University of British Columbia, Vancouver, British Columbia, Canada; 3 Department of Microbiology and Immunology, the Michael Smith Laboratories and the Biomedical Research Centre, University of British Columbia, Vancouver, British Columbia, Canada; 4 Department of Zoology, the Michael Smith Laboratories and the Biomedical Research Centre, University of British Columbia, Vancouver, British Columbia, Canada; City of Hope Medical Center and Beckman Research Institute, United States of America

## Abstract

**Background:**

Therapeutic intervention in many neurological diseases is thwarted by the physical obstacle formed by the blood-brain barrier (BBB) that excludes most drugs from entering the brain from the blood. Thus, identifying efficacious modes of drug delivery to the brain remains a “holy grail” in molecular medicine and nanobiotechnology. Brain capillaries, that comprise the BBB, possess an endogenous receptor that ferries an iron-transport protein, termed p97 (melanotransferrin), across the BBB. Here, we explored the hypothesis that therapeutic drugs “piggybacked” as conjugates of p97 can be shuttled across the BBB for treatment of otherwise inoperable brain tumors.

**Approach:**

Human p97 was covalently linked with the chemotherapeutic agents paclitaxel (PTAX) or adriamycin (ADR) and following intravenous injection, measured their penetration into brain tissue and other organs using radiolabeled and fluorescent derivatives of the drugs. In order to establish efficacy of the conjugates, we used nude mouse models to assess p97-drug conjugate activity towards glioma and mammary tumors growing subcutaneously compared to those growing intracranially.

**Principal Findings:**

Bolus-injected p97-drug conjugates and unconjugated p97 traversed brain capillary endothelium within a few minutes and accumulated to 1–2% of the injected by 24 hours. Brain delivery with p97-drug conjugates was quantitatively 10 fold higher than with free drug controls. Furthermore, both free-ADR and p97-ADR conjugates equally inhibited the subcutaneous growth of gliomas growing outside the brain. Evocatively, only p97-ADR conjugates significantly prolonged the survival of animals bearing intracranial gliomas or mammary tumors when compared to similar cumulated doses of free-ADR.

**Significance:**

This study provides the initial proof of concept for p97 as a carrier capable of shuttling therapeutic levels of drugs from the blood to the brain for the treatment of neurological disorders, including classes of resident and metastatic brain tumors. It may be prudent, therefore, to consider implementation of this novel delivery platform in various clinical settings for therapeutic intervention in acute and chronic neurological diseases.

## Introduction

Neurological diseases associated with cancers, inborn errors of metabolism (such as lysosomal storage diseases), infectious diseases and aging create a significant social and economic burden. Demographics predict that, in the aging populations of the economically developed world, the incidence of cardiovascular disease, dementias and cancer will continue to dramatically increase over the next 20 years. Despite advances in molecular screening technologies, which have spawned new drug targets and therapeutic candidates for neurological diseases, these technologies have yet to find conduits for successful clinical application due in part to disease complexity, the individual variability within the human population and the poor early diagnosis of disease. However, the most serious impediment to pharmaceutical treatment is inefficient delivery of drugs to the disease-affected brain tissue, due to limitations and restrictions dictated by the existence of the blood-brain barrier (BBB) [1]. Designing efficient ‘vectors’ (antibodies, protein carriers, viruses, nanoparticles) to navigate and deliver therapeutics across the BBB in a controlled and non-invasive manner remains one of the key goals of drug development for brain diseases.

The BBB is found in all vertebrates and is selectively permeable. The BBB endothelial cells are known as the “gatekeepers of the brain” and the barrier is formed by the presence of high resistance tight junctions that fuse brain capillary endothelia into a continuous tubular cell layer separating blood from the brain. In addition to keeping unwanted substances out, the BBB helps retain brain-synthesized compounds, such as neurotransmitters. Fine structural differences exist between the endothelia of the brain capillaries and endothelia in other capillaries. These include tight junctions between adjacent endothelial cells [2], a paucity of pinocytotic vesicles [3], [4] and a lack of fenestrations (perforations) [5]–[7]. The cerebral endothelium forms tighter junctions than other endothelia that are characterized by greater electrical resistance and contain specific proteins, including enzymes and transporters, whose expression appears to be augmented in comparison to other endothelia [4], [8]–[11]. The physiochemical properties of the penetrating substance largely determine whether or not it can penetrate or be transported across the BBB. In general, small hydrophobic solutes can readily cross the BBB, while hydrophilic substances are selectively transported across the barrier by specific trans-endothelial membrane carrier proteins. Thus, many substances in the blood cannot transit the BBB since they are not compatible with the resident carrier systems.

The tightly sealed brain vasculature forming the BBB precludes virtually all systemically injected macromolecular drug compounds and most hydrophilic drugs from entering the brain. Furthermore, efflux transporter proteins expressed at the BBB, such as P-glycoprotein (Pgp1) [1], present significant problems for the treatment of brain tumors with current chemotherapeutics [12] as they act to pump small hydrophobic chemotherapeutic agents out of the brain. Many methods developed to enhance the delivery of drugs to treat brain tumors have failed to provide significant improvements to long-term survival [13]–[25]. Radical methods to transiently increase the permeability of the BBB allowing diffusion of injected drugs into the brain [26] [27] [28] cause damage by uncontrolled entry of the blood constituents into the brain. Thus, specific drug design is limited by factors such as lipid solubility, charge, molecular weight and the antiport action of specific transporters [29], [30]. A large assortment of drugs conjugated to peptides [26], [31]–[33], to proteins [1], [31], [32], or to antibodies [28], [34], [35] able to bind to receptors expressed on the luminal surface of the BBB have been investigated. MRC OX26, a monoclonal antibody against the rat transferrin (Tf) receptor [36], [37] has been used to study transport across the BBB [35], [38], [39]. Although partially effective for transport into the brain, the use of antibodies such as MRC OX26 appears to be limited due to saturation of the receptor with antibody, low dissociation rate of the antibody (and indirectly its potential payload of therapeutic compounds), and recycling of the receptor back to the blood [40]. Furthermore, hypersensitivity resulting in hyperimmunity against the foreign monoclonal antibody carrier may also limit repeated treatments with the antibody-drug conjugates. In addition, many targeted receptors are widely expressed in other tissues resulting in potential toxicity [36], [37], [41]. Finally, lost in the considerable data on potential transporters, carriers and delivery systems it is an unfortunate realization that, at present, none of these approaches are efficacious in treating diseases that lay beyond the BBB. Thus, novel approaches are required to increase the survival of patients with acute and chronic neurological diseases.

Here we have focused on the iron binding protein p97 (melanotransferrin), a protein closely related to Tf and lactoferrin (Lf) [42]. As a result of alternative splicing, p97 exists in both a soluble form and a cell surface GPI-linked form [43]. However, in normal brain it appears to discretely localize on the surface of endothelial cells and transiting through brain capillary endothelium [10], [44]. Studies on the structure and function of p97 suggest it might be an ideal carrier for transport of drug conjugates into the brain [31], [33], [34], [37], [45], [46]. Recombinant p97 is actively transported across the BBB in an *in-vitro* model [47] of BBB trancytosis [48], with a transport rate 10 to 15 times higher than that of either Tf or Lf. [48]. In addition, studies on its biodistribution support the concept that p97 injected intravenously preferentially distributes in brain tissue [39], [49]. Furthermore, the mechanism of transport is likely receptor-mediated transcytosis, possibly involving a member of the low-density lipoprotein receptor–related protein family (LRP) [49]–[52]. The p97 protein exists at low serum concentrations (under 7.5 ng/mL = 0.08 nM in healthy adults) suggesting that the native protein should not significantly block the binding of exogenously-injected p97 conjugates from reaching the receptors in the BBB. Furthermore, the transcytosis of p97 is likely to allow piggybacked therapeutic compounds to bypass the efflux transporter Pgp-1. Thus, based on these collective properties, we hypothesized that p97 may be an attractive new candidate as a drug delivery vector. Here we test this hypothesis in a mouse model by assessing the chemotherapeutic activity of p97-drug conjugates within the brain in comparison with unconjugated drugs. We demonstrate that intervention with p97-drug conjugates provides a marked improvement over current ineffective chemotherapies for cancers of the brain. The p97 protein, therefore, is the first carrier that efficiently transports chemotherapeutic agents across the BBB that therapeutically modulates disease within the brain. Thus, p97 may have wide utility as a drug delivery vehicle for the treatment of a variety of inoperable neurological conditions.

## Methods

### Cells and Animals

C6 rat glioma (ATCC CRL-2199) and ZR-75-1 human mammary tumor (ATCC CRL-1500) cells were cultured in DMEM supplemented with 10% heat inactivated fetal bovine serum (FBS) at 37°C in 5% CO_2_ in air atmosphere. BHK TK^-^ ts13 cells transfected with a full-length human p97 cDNA [53] were cultured in DMEM supplemented with 10% FBS, 20 mM HEPES, 2 mM L-glutamine, 0.08 mM zinc sulphate and 500 µM methotrexate [53]. Soluble p97 was affinity purified as previously described [53], its concentration determined a quantitative antibody sandwich assay [54] and its purity determined by SDS-PAGE analysis [54]. Female NSWNU (m) Swiss nu/nu mice aged 6–8 weeks were used for tumor models. Both male and female C57Bl/6 mice, aged 6 to 8 weeks, were used in all other studies. All procedures involving mice were in accordance with guidelines set by the UBC Animal Care Committee, which states that mice must be euthanized if they lose more than 20% of their starting weight or if the tumor size should exceed 5% of the animal's normal weight.

### Microscopy

Holo-human p97, holo-human transferrin (huTf), and holo-murine transferrin, mTf, (Sigma) were labeled with Alexa Fluor 488 (Alexa 488) protein labeling kit (Molecular Probes, MP) for confocal microscopy or using a DIG protein labeling kit (Boehringer-Mannheim) for electron microscopy (EM). Labeled or unlabeled holo-proteins (0.3 mg) were injected into the tail vein. Human Tf was used in order to allow differentiation from endogenous murine Tf. After 1 h, mice were perfused through the left ventricle with PBS, followed by 4% paraformaldehyde in PBS. The brains were immediately dissected, paraffin embedded and later sectioned. Brains were stained with either the anti-human p97 monoclonal antibody, L235 [55] or the rabbit anti-human Tf antibody (Research Diagnostics). Antibody binding to these tissue sections was subsequently visualized with goat anti-mouse Ig Alexa 488 or goat anti-rabbit Ig Alexa 488 (Molecular Probes).

Fluorescent PTAX (Oregon Green® 488 paclitaxel; Molecular Probes) was diluted in buffer and 100 µg were injected *i.v.* into each mouse. In addition, Oregon Green® 488 was conjugated to p97 as directed by the supplier, and 0.2 ml of the conjugate containing 100 µg of the dye was injected *i.v*. Injection was repeated 3 times during the day and mice were sacrificed by the end of the day. Then organs were harvested and analyzed later by fluorescent microscopy.

For electron microscopy (EM), 0.4 mg of p97 and p97-DIG labeled with 13 nm and 5 nm gold particles (British Biocell) respectively, were injected simultaneously into the tail vein. After 1 hr, mice were perfused and brains processed for EM [39]. Thin sections were stained with anti-DIG antibody (British Biocell) or visualized by gold enhancement (Nanoprobes).

### Stability of ^125^I-p97 in Serum and Urine

Urine and serum samples were centrifuged at 9000× g for 15 min at 4°C. Samples were reconstituted in SDS-PAGE buffer containing a final concentration of 1% SDS and were heated to 95°C for 10 min before gel separation. For reducing conditions, 2% ß-mercaptoethanol was included in the SDS-PAGE buffer. SDS PAGE gels (12%) were run with Tris-glycine-SDS buffer and after electrophoresis were dried and exposed to Kodak XAR Film for 2–4 days.

### Uptake of ^125^I-p97 into the Brain

To measure the brain uptake of ^125^I-p97, mice were each given approximately 4 pmol of ^125^I-p97 (0.026 µg in 1.8 µCi) in 200 µl of injection solution 14.2 µg/mouse lactated Ringer's solution or 0.25 M phoshate buffered saline (PBS) containing 1% bovine serum albumin (BSA) through the tail vein at time 0 with a Hamilton syringe. After the radiolabeled p97 has been circulating in the blood for 2 hours, organs were collected for radioactivity measurement. The serum and brain samples were collected and the levels of radioactivity were measured.

### Multiple Regression Analysis of Entry of ^125^I-p97 into the Brain

^99^Tc-albumin with comparable radioactivity was included in this injection solution as a vascular marker. At 5, 10, 15, 20, 25 and 30 min after injection, blood was collected from the right carotid artery and the mice (*n = *5 for each time point) were immediately decapitated. The radioactivity in 50 µl of serum and of weighed brain samples were measured by a dual channel γ-counter. This study provided information on the brain uptake of ^125^I-p97 at certain time points and further served as the control for the concurrent investigation into whether the brain entry of ^125^I-p97 was saturable. Self-inhibition of ^125^I-p97 brain uptake was tested by inclusion of unlabeled p97 (1∶ 500 and 1∶ 1000) in the injection mixture (7.1 mg/mouse; *n = *3 for each time point). Distribution of albumin in different organs was measured in similar fashion as the p97. To correct for the decrease of ^125^I-p97 concentration from blood with time, exposure time (*t*), which his the integral serum radioactivity at time 0 to time *t* divided by the serum radioactivity at time *t* [56], [57], was calculated. The organ/serum ratio of radioactivity (ml/g) was plotted against exposure time. The slope of the linear part of this regression line represents the influx rate, and the intercept at time 0 is the initial volume of distribution of ^125^I-p97 in the organs for each group. The half-time disappearance was determined from the regression line obtained from the plot of the logarithm of brain radioactivity against time. The unidirectional influx constant (*K_i_*), expressed in µl/g-min, and the apparent volume of distribution of the brain (*V_i_*), in µl/g were determined from the linear portion of the relationship between brain/serum ratios and t with the equation; brain/serum ratio [(cpm/g brain)/cpm/µl/g serum)] = *K_i_* (*t*)+*V_i_*.

### Capillary Depletion Method to Determine the Brain Compartmental Distribution of ^125^I-p97

A capillary depletion procedure [58], [59] was employed to separate the cerebral capillaries from the brain parenchyma. Blood was collected from the carotid aorta of mice (n = 5) at 60 min following the *i.v.* tail injection of 3.6 mCi ^125^I-p97. Subsequently, the jugular veins were cut, the descending aorta was blocked and the mice received an intracardial perfusion of injection solution before decapitation. For each mouse, the brain was dissected, weighed, homogenized by three passages through a 20 gauge syringe in 2 ml of 1 mg/ml collagenase/dispase. The mixture was incubated at 37°C for 30 to 40 min and then washed one time in PBS. The material was resuspended in 6 ml PBS and equally split into 3 Corex tubes. Two ml of 26% dextran was added to each tube to enable pelleting of the brain capillaries. The samples were mixed and then centrifuged at 5400 *g* for 15 min at 4°C. The resulting pellet (cerebral capillaries component) and the supernatant (brain parenchymal/interstitial fluid space) were carefully separated and the percent contamination of the supernatant with vasculature was assessed by measuring the specific activity of the endothelial marker, γ-glutamyl transpeptidase. Radioactivity of each sample was counted with a dual channel γ-counter. The ratios of radioactivity of ^125^I-p97 in the supernatant (parenchyma) or pellet (capillary) over serum were calculated, and the contamination of the vascular component was further corrected by subtraction of the^ 99^Tc-albumin ratios.

### Synthesis and Analysis of p97-PTAX Conjugates

Conjugation of PTAX to p97 followed the procedures described previously [60]. The MSR of p97 was determined by absorbance analysis and was determined to be similar to transferrin, which has a conjugate to carrier ratio of 5 PTAX molecules per p97 molecule [60]. The stability of the conjugates in buffer and in mouse sera was measured by HPLC at room temperature (RT) over a period of 1 to 120 hrs.

### Extraction Method for PTAX analysis

Tissue extraction was performed by a modification of the method of Sparreboom *et al.* [61] and the PTAX metabolites were identified according to the method of Royer *et al.* [62]. Thawed tissue was Dounce homogenized, then pipetted into a glass tube and made up to 1 ml with 4% BSA solution. Tissue samples underwent diethyl ether extraction and solid-phase extraction (SPE). Plasma samples consisted of 250 µl of thawed murine plasma and underwent SPE only. Samples with less than 250 µl volume were filled up with human citrate-phosphate-dextrose/plasma. To each sample 10 µl of 50 µM docetaxel solution (Taxotere diluted with absolute ethanol; Rhone-Poulenc Rorer) were added as internal standard. HPLC analysis was performed on an Agilent HPLC apparatus. A stainless steel (125×4 mm) analytical column equipped with a guard column (4×4 mm), both packed with 5 µm LiChrospher 100 RP-18 material, was cooled to 33°C. The mobile phase consisted of acetonitrile/methanol/0.2 M ammonium acetate buffer, pH 5.0, 38∶ 10.5∶ 51.5 (vol/vol). UV detection was performed at 227 nm, and washing gradient was applied after each run (additional 35% vol/vol acetonitrile). Injection volumes were either 50 or 100 µl. For calibration, PTAX and docetaxel stock solutions in anhydrous ethanol, stored at −20°C, were diluted with mobile phase (1∶ 100) on the day of analysis. The method of analysis was validated for murine plasma, brain, liver, and kidney samples. Correlation coefficients for PTAX calibration curves in the concentration range 50–1000 nM were greater than 0.999. Recovery rates for PTAX ranged from 70 to 85%, depending on PTAX concentration and the type of tissue.

### Brain Uptake Trials Involving p97-PTAX

Delivery of PTAX to brain was examined by the following procedure: mice (n = 10) received 5 injections of 8 mg/kg of PTAX over one week. Before organ dissection, mice were anesthetized (ketamine-HCl 100 mg/kg, xylazine 10 mg/kg) and a 300 µL sample of blood removed by cardiac puncture. The left atrium was snipped with scissors and the mouse was perfused with heparinized saline from a peristaltic pump through a 27 gauge needle, thereby removing all blood. PTAX concentration was measured in brain and serum. In 5 additional mice, delivery and stability of PTAX was measured after a single injection and the blood concentration and stability of the conjugate (p97-PTAX) was measured at different time points (0.25, 0.50, 1, 1.5, 2, 6 and 12 hrs; refer to Table 1) based on the free amount of PTAX present in serum.

**Table 1:** Concentration and Stability of p97-PTAX *in vitro* and *in vivo*.

Concentration of p97-PTAX in Buffer (ng/ml)		Concentration of p97-PTAX in Serum After injection (nmol/kg)		Concentration of p97-PTAX in Brain After Injection (nmol/kg)	
**1 hr**	80±2.1	**15 min**	2.0±1.37	**15 min**	0.06±0.001
**5 hrs**	100±9.1	**30 min**	0.6±0.14	**30 min**	0.007±0.001
**24 hrs**	120±8.4	**1 h**	1.0±0.8	**1 h**	0.01±0.003
**48 hrs**	100±7.8	**1.5 h**	1.5±0.2	**1.5 h**	0.015±0.0002
**72 hrs**	80±**2.4**	**2 h**	2.0±**0.2**	**2 h**	0.01±0.004
**94 hrs**	30±**4.6**	**6 hrs**	0.8±**0.42**	**6 hrs**	0.09±0.006
**120 hrs**	10±5.8	**12 hrs**	0.2±0.01	**12 hrs**	0.07±0.003

Concentration of p97-PTAX conjugate in buffer at RT, in serum after *i.v.* injections and in mouse brain after *i.v.* injection. Free PTAX could not be detected in brain.

Sample number in buffer and serum, *n* = 5; in brain, *n* = 10.

+/− are standard deviations.

### Synthesis and Analysis of p97-ADR Conjugates

Conjugation of ADR [(8S,10S)-10-(4-amino-5-hydroxy-6-methyl-tetrahydro-2H-pyran-2-yloxy)-6,8,11-trihydroxy-8-(2-hydroxy-acetyl)-1-methoxy-7,8,9,10 tetrahydrotetracene-5,12-dione] to p97 was achieved by cross-linking p97-N-succinimidyl S-acetylthioacetate (p97-SATA) and ADR-succinimidyl 4-[N-maleimidomethyl]-cyclohexane-1-carboxylate (ADR-SMCC) derivatives. SATA (Pierce Chemical Co, St. Louis MO) and the p97-SATA were prepared according to instructions supplied by the supplier. Activated ADR-SMCC (>96% pure) was synthesized by Albany Molecular Research Inc. The conjugation of deacylated p97-SATA to the activated ADR-SMCC was achieved by combining these compounds at 4°C overnight. Conjugates (p97-ADR) were purified on 5 mL D-Salt Excellulose (Pierce Chemical Co.) desalting columns equilibrated with PBS. Purified p97-ADR was assessed by SDS-PAGE, anion exchange chromatography and Western blot analysis using the primary anti-human p97 monoclonal mouse antibody L235 [63]. Individual conjugates were designated as SYN002, SYN018, SYN019, and SYN020. The molecular substitution ratio (MSR) or moles of ADR bound to moles of p97, were determined by absorbance analysis from standard curves established for ADR and p97 at 486 and 280 nm wavelength. The two conjugates were found to have 4 to 7 molecules of ADR per molecule of p97 at an ADR concentration of 17 to 60.6 µg/ml.

### Radiolabeling of Compounds

p97, Lf, and p97-ADR conjugates were iodinated using a standard chloramine T protocol [52]. The specific activity was calculated from the radioactivity of the precipitable fraction after trichloroacetic acid (TCA) precipitation. Bovine serum albumin (BSA) in the form of ^99^ Tc-albumin was purchased from Amersham (now GE Healthcare) and had a specific activity of 100 Ci/g**.** [^14^C]-labeled ADR was purchased from Nycomed.

### Stability and Quality of p97-ADR Conjugates

To determine the stability of the p97-ADR conjugate, SYN002 was prepared with [^14^C]-labeled ADR. Aliquots of 200 µL were added to 1 mL of mouse sera and incubated at 37°C. Over a period of 18 hr, 25 µL aliquots were removed and the CPM of the TCA-precipitable fraction was determined. The samples were run on 12% SDS-PAGE gels as described above and protein positions were visualized after exposure to Kodak XAR film for 25 days.

### Treatment of Mice Bearing Subcutaneous Tumors

C6 glioma tumors were established subcutaneously in mice by injecting 50 µL of 1×10^5^ rat C6 glioma cells into the right flank. Groups of 3–10 mice were treated (detailed in Table 2), starting shortly after the tumor cells, with p97-ADR conjugate (SYN002 - 0.0625 mg/mL ADR), ADR (0.0625 mg/mL in PBS) or PBS alone by 5–10 tail vein injections (8 µL/g body mass each time) over a period of 14–25 days for a total free or conjugated ADR at 4 mg/kg. Tumor size was monitored over 19 days. At the end of the trial, serum was collected and analyzed for two markers of cardiotoxicity: creatine kinase (CPK) and lactate dehydrogenase (LDH)[64], [65]. Control treatments were either PBS or ADR alone.

**Table 2:** Injection Schedule of ADR-conjugates in Different Mouse Models.

Trial	*n*	Tumour Cell type	Tumour location	Treatment	Injection schedule (Day)	Total ADR (mg/kg)
**1**	9	C6	Subcutaneous	PBS	3,4,5,6,10,11,12,13 (8)	0
	9	C6	Subcutaneous	ADR	3,4,5,6,10,11,12,13 (8)	4
	9	C6	Subcutaneous	SYN002	3,4,5,6,10,11,12,13 (8)	4
**2**	10	ZR-75-1	Intracranial	PBS	3,4,5,6,7,10,11,12,13,14 (10)	0
	10	ZR-75-1	Intracranial	ADR	3,4,5,6,7,10,11,12,13,14 (10)	20
	10	ZR-75-1	Intracranial	SYN002	3,4,5,6,7,10,11,12,13,14 (10)	5.5
**3**	10	C6	Intracranial	PBS	1,3,7,10,14 (5)	0
	10	C6	Intracranial	SYN018	1,3,7,10,14 (5)	0.49
**4**	10	C6	Intracranial	PBS	2,9,17,20,25 (5)	0
	10	C6	Intracranial	ADR	2,9,17,20,25 (5)	20
	10	C6	Intracranial	SYN002	2,9,17,20,25 (5)	2.75
**5**	2	N/A	N/A	BSA	(1)	0
	2	N/A	N/A	Lf	(1)	0
	5	N/A	N/A	p97	(1)	0
	3	N/A	N/A	SYN019	(1)	0.1
	3	N/A	N/A	SYN020	(1)	0.1

Injection schedule indicates the number days after the tumor implant when the single injections of the specific treatments were administered. The number in the brackets indicates the total number of injections. All injections were *i.v.* made via the tail vein with the exception of Trial 4, which used intra-jugular injections.

### Treatment of Mice Bearing Intracranial Tumors

Mice were anaesthetized with ketamine (100 mg/kg) and xylazine (10 mg/kg). A motorized injector delivered 4×10^5^ C6 glioma or 1×10^6^ ZR-75-1 cells in 5 µL PBS at rate of 1 µL/min from a 25 µL Hamilton syringe through a 27 gauge needle to a position located 3 mm below the surface of the skull, 3 mm in front of coronal suture and 3 mm to the right of midline. The needle was removed slowly two minutes after the completion of the injection. The site was wiped with an alcohol soaked swab and sealed using sterile bone wax, and the scalp incision closed with sterile clips. Mice bearing intracranial C6 glioma were treated with repeated injections (8 µL/g body mass - jugular vein) of p97-ADR (SYN002 - 0.06875 mg/ml ADR) for a total dose of 5.5 mg/kg ADR, or free ADR (0.5 mg/ml ADR in PBS) for a total dose of 20 mg/kg, or PBS alone.

In Trial 2, the effectiveness of SYN002 conjugate against intracranial ZR-5-1 mammary tumors was assessed by repeated tail vein injections of 8 µL per g body weight solutions containing PBS, ADR (0.25 mg/ml ADR in PBS) or SYN002 (0.06875 mg/ml ADR). The dosing regime is outlined in Table 2 and carried out over a period of 15 days to a total of 20 mg/kg free ADR or 5.5 mg/kg conjugated ADR. Survival was reported as the percentage of total mice receiving an intracranial tumor. The data were represented as Kaplan-Meier survival curves. Mean and median survival in days since tumor implantation were calculated for each treatment. Efficacy of the conjugate and free ADR were determined as a percent increase of mouse survival time as compared to mice given PBS as control.

### Brain Uptake Trials Involving p97-ADR

Mice received tail vein injections of ^125^I-p97-ADR conjugates (SYN019, SYN020), ^125^I-p97, ^125^I-BSA, or ^125^I-Lf (8 µl/g body mass, 3 µg protein/ml). One hour after injection, mice were anesthetized (ketamine-HCl 100 mg/kg, xylazine 10 mg/kg) and a 300 µL sample of blood removed by cardiac puncture. The left atrium was snipped with scissors and the mouse was perfused with heparinized saline from a peristaltic pump through a 27 gauge needle. After perfusion, the brain was removed and CPM per gram of tissue determined using a Gamma counter (CobraII, Packard, IL).

### Statistical Analysis

Means are reported with their standard errors. Groups were compared by one-way analysis of variance (ANOVA) followed by Duncan's multiple or Tukey's range test. For regression analysis, the least squares method was used, as well as the difference between the slopes of regression lines.

## Results

### Study design

The biodistribution of intravenously injected p97 vector, free drug, or p97-drug conjugates were examined initially. The modulation of growth of representative classes of tumors growing in peripheral tissues outside the BBB (subcutaneously) and growing cloistered behind the BBB (intracranially) were examined including resident (rat C6) glioma and metastatic (human ZR-25-1) mammary tumor cells. Both tumor types present with reliable characteristic of growth, which parallels patterns witnessed in the equivalent diseases in humans. They both have 100% take rates in nude mice and reproducibility in survival patterns [66], [67]. We chose to examine the therapeutic effects of ADR and p97-ADR conjugates rather than PTAX and p97-PTAX because C6 is resistant to the cytostatic activity of PTAX [68]. We recorded tumor size and mouse mortality in groups of nude mice inoculated either subcutaneously or intracranially with aggressively growing xenogeneic tumor types (rat glioma and human mammary tumors) which were then treated with p97-ADR, free drug or saline controls.

### Stability and Biodistribution of p97

**Figure 1:**
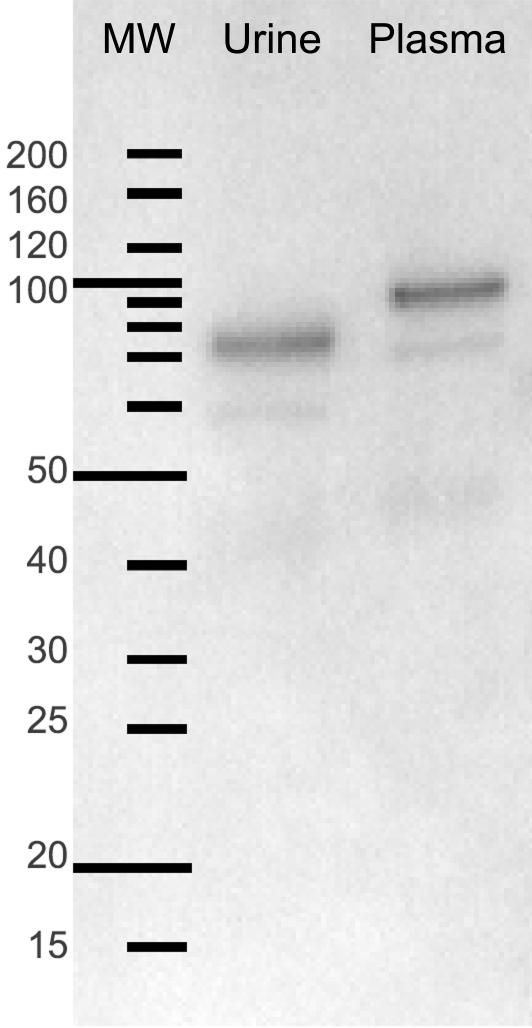
Stability of p97 in the mouse 1 hr. after intravenous injection. Iodinated p97 protein is found intact in plasma after 1 h post-*i.v.* injection, but appears to undergo a cleavage event prior to appearance in urine.

**Figure 2:**
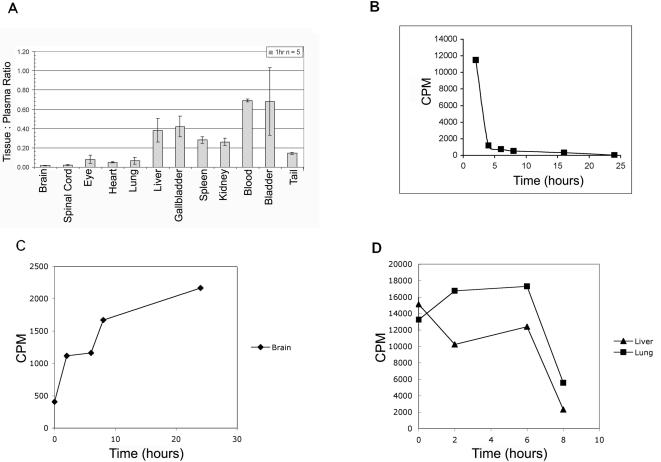
Distribution of p97 in tissues and organs after intravenous injection. (a) Tissue plasma ratios of iodinated p97, normalized by organ mass, in a variety of organs at one hour post-injection, including: whole brain, spinal cord, eye, heart, lung, liver, gall bladder, spleen, kidney, blood, bladder, and tail, *n* = 5 mice. (b) The plasma concentration radioactivity, normalized by plasma mass, of ^125^I-p97 after *i.v*. injection decreases over time. The figure represents data from one mouse. (c) The radioactivity, normalized by plasma mass concentration, of ^125^I-p97 after *i.v*. injection increases in brain over time. The figure represents data from one mouse at each time point.

**Figure 3:**
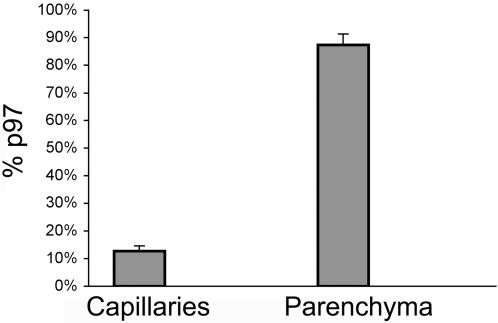
Intravenously injected p97 is able to cross the BBB and enter the brain. At 6 h post-injection greater than 80% of the p97 in the brain is found in the parenchyma rather than the capillary blood vessels.

The stability of iodinated p97 was verified at room temperature in buffer and in serum samples taken at different time points after injection. Plasma analysed by SDS-PAGE at 1 hour post-*i.v.* injection showed the great majority of ^125^I remained at the p97 MW position (Figure 1a). Its appearance in urine was accompanied by a proteolytic cleavage event (Figure 1). One hour after injection, ^125^I-p97 levels in the blood, kidney, bladder, liver, spleen, gallbladder, eye, heart and lung were all higher than in the central nervous system (Figure 2a). The initial uptake of p97 in the kidney, liver and spleen increased rapidly within minutes of injection and then decreased within the subsequent few hours. After 1 hr, p97 was found to accumulate in the brain while its level in all other organs decreased. The decrease in p97 concentration in plasma over time is shown in Figure 2b, whereas 10 minutes after injection of iodinated p97, it begins to accumulate in the brain (Figure 2c). After 6 h, the majority of ^125^I-p97 associated with the brain was localized to the brain parenchyma (83%), and not the blood vessels (Figure 3). Unlike the other organs, the brain continued to accumulate p97 at 24 hours after injection. After 24 hrs, the total accumulation of p97 in the brain reached 1–2% of the injected dose.

### Entry of p97 into the Brain via a Receptor-Mediated Process

**Figure 4:**
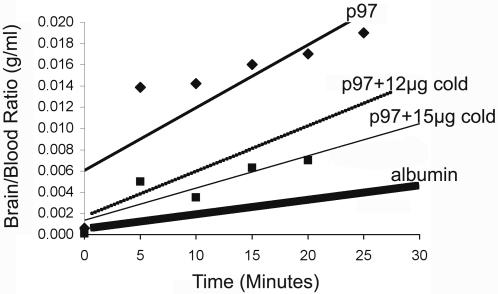
Multiple time^-^regression analysis of the rate of p97 entry into the brain. The entry of ^125^I-p97 into the brain, measured by the brain/serum ratios of radioactivity (µl/g), was linear when plotted against exposure time. Addition of unlabeled p97 dose responsively inhibited the entry of the radiolabeled ^125^I p97 protein. The influx rate (*K*_i_) for each group was found to be as follows: (i) ^125^I-p97 only: 2.02±0.28 ml/g-min; (ii) ^125 I-^p97 (plus 12 µg of cold p97): 0.45±0.08 ml/g-min; and (iii) ^125^I-p97 (plus15 µg of cold p97): 0.16±0.19 ml/g-min. The difference among the slopes of the regression lines was statistically significant, p<0.0001. The graph represents one mouse.

Supporting the hypothesis that p97 enters the brain through a receptor-mediated process on the BBB, we have found that the rate of uptake into the brain of *i.v.* injected ^125^I-p97 can be inhibited in a dose related manner by an excess of unlabeled p97 (Figure 4), supporting previously published data [52]. This “cold block” signifies competition for a receptor to the point of saturation. Competition with an excess of nonradiolabeled p97 in other organs such as the liver, kidneys and lungs is very low (data not shown), suggesting that p97 is not taken up in these organs in a receptor-mediated fashion. It also suggests that the brain expresses a higher concentration of receptors for p97 than these other organs. Using multiple-time regression analysis [57], [69] we found that the influx constant (*K_i_*) is higher for p97 (Figure 4) than that for albumin [70], which crosses the BBB through extracellular pathways, again suggesting the presence of a receptor mediated system of ^125^I-p97 transport. The entry of ^125^I-p97 into brain, measured by the brain/serum ratios of radioactivity (µl/g), was linear when plotted against exposure time. A dose-related self-inhibition of the *K_i_* for ^125^I-p97 brain uptake was achieved by co-administrating an excess of unlabeled p97 protein. The brain influx rate (*K_i_*) for each group is as follows: (a) ^125^I-p97 only: 2.02±0.28 ml/g-min; (b) ^125^I-p97 plus 12 mg of p97: 0.45±0.08 ml/g-min; and (c) ^125^I-p97 plus 15 mg of p97: 0.16±0.19 ml/g-min. The difference in the regression lines slopes was statistically significant [F(2,17) = 21.8, P<0.0001]. The finding that the entry of p97 into the brain can be significantly inhibited by unlabelled p97 suggests that this may involve a receptor-mediated process. The addition of unlabeled p97 did not change the entry rate for ^99^Tc-albumin (overall *K*_i_ = 20.1±0.03 ml/g-min).

### Microscopic Visualization of p97-Conjugates in the Brain

**Figure 5:**
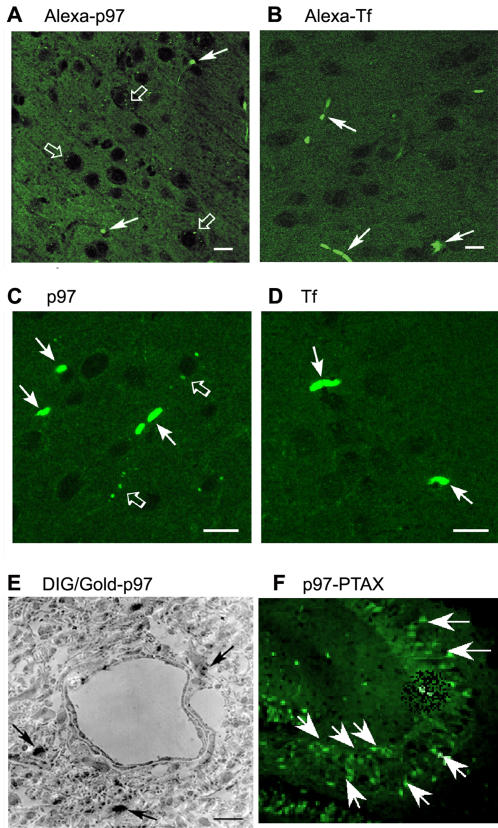
Visualization of p97 and Tf uptake in the brain. (a) Alexa 488 labeled holo-p97 and (b) Alexa 488-holo-Tf appear in the brain after one hr following injection in mice. Although p97 and Tf can be seen in the microvessels of respective mice (solid arrows), p97 appears to transcytose the BBB more efficiently than Tf and exhibits a punctate distribution in the cytoplasm of cerebral cortical cells (open arrows). Immunohistochemistry of (c) holo-p97 and (d) holo-Tf in the brain one hr after intravenous injection, using respective antibodies, show that although the two proteins are seen in the microvessels of respective mice (solid arrows), p97 appears to transcytose the BBB more efficiently than Tf and exhibits a punctuate distribution in the brain parenchyma (open arrows). Scale bar represents 5 µm. (e) After DIG-labeled p97 was injected into a mouse, the brain was harvested, sectioned, and the p97 localized with colloidal gold conjugated anti-DIG antibody and visualized by gold enhancement. Although parenchymal structures are weakly fixed, this EM shows that DIG conjugated p97 crosses the intact BBB and can be seen in the brain parenchyma. (f) Fluorescent PTAX is clearly shown within hipocampal brain sections of mice after the 5^th^ injection (solid arrows).

Drug carriers that efficiently piggyback drugs into the brain after peripheral administration would be expected to first localize to the brain then rapidly translocate within the parenchyma, and then perhaps eventually emerge in the cerebrospinal fluid. Thus we examined if (i) fluorescence-labeled conjugates of p97 or (ii) unlabeled could efficiently traverse the BBB and appear in the parenchyma of the brain. One hour after *i.v.* injection, p97-Alexa 488 conjugate (Figure 5a) and p97 (Figure 5c) could be seen both in the lumen of brain microvasculature and within cortical cells by fluorescence microscopy. Fluorescence in cortical cells exhibited a punctuate distribution in the cytoplasm but not in the cellular processes. This staining pattern was in contrast to fluorescence associated with Tf-Alexa 488 (Figure 5b) and Tf (Figure 5d). Punctate cytoplasmic staining in cortical cells was not observed for Tf-Alexa 488 or Tf, despite strong staining in the microvasculature similar to that observed for p97. This is consistent with previous observations that p97 can effectively traverse the BBB. The integrity of the BBB following *i.v.* injection of p97-DIG conjugate (Figure 5e) was examined using EM. Specific staining for p97-DIG was observed in the brain parenchyma abluminal to the microvasculature. Despite weak fixation, the BBB was demonstrated to be intact, indicating that p97-DIG crossed into the brain without disruption of the BBB. Furthermore, p97-PTAX was clearly identified in brain parenchyma (Figure 5f), whereas free PTAX could not be seen in brain but was visualized in other organs such as heart and liver (not shown). These data indicate that p97 can transport a wide variety of molecular compounds across the BBB and that the drugs conjugates remain intact following delivery to the brain.

### Stability and Biodistribution of ADR and PTAX Conjugates

The stability of p97-PTAX conjugate was verified in buffer and in serum samples at ambient (room) temperature, taken at different time points after injection by SDS-PAGE analysis and HPLC analysis. As determined by HPLC analysis, the conjugate stored in buffer was stable for over 24 hrs at room temperature and in the blood for up to 4 hours (Table 1). The p97-PTAX conjugate (Figure 5f) and its metabolites were detected in brain tissue but free PTAX was not detected in the brain (Table 1).

**Figure 6:**
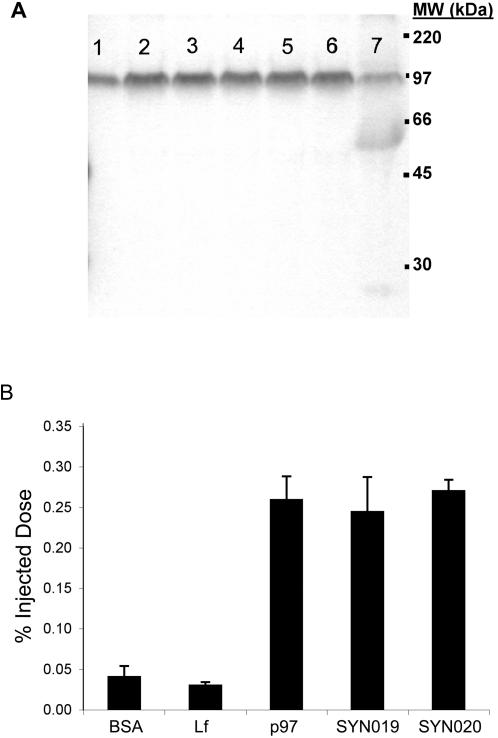
The stability and distribution of p97 and p97-conjugates in the brain of mice. (a) p97-ADR[^14^C] appears to be stable in mouse serum over time. Lanes. 1 = 0 (minutes) and 90% (TCA precipitable counts), 2 = 10 min/92%, 3 = 20min/94.7%, 4 = 30min/90.5%, 5 = 40min/83.9%, 6 = 60min/93.9%, and 7 = 18 hours/83.3%. The position of the [^14^C] molecular weight markers are as indicated. (b) A comparison of protein and protein conjugates crossing the BBB in mice 1 h after tail vein injection. CPM per g of brain tissue as a percentage of the total CPM injected. The albumin (BSA) (n = 2) and lactoferrin (Lf ) (n = 2) show low levels while p97 (n = 5) and both p97-ADR conjugates SYN019 (n = 3) and SYN020 (n = 3) are significantly higher at one hour post injection.

The stability of the p97-ADR conjugates was directly assessed by analyzing tissues and body fluids post injection. p97-[^14^C]ADR conjugate was stable in mouse serum for at least 1 h, with some degradation observed after 18 h (Figure 6a). Protein-associated radioactivity after TCA precipitation remained above 80% for the duration of the experiment. The MSR for the p97-ADR conjugates ranged between 5 and 6. The brain uptake of ^125^I-p97 and ^125^I-p97-ADR was compared to ^125^I-BSA and ^125^I-Lf one hour after a single bolus tail vein injection (Figure 6b). Uptake of ^125^I-p97 and ^125^I-p97-ADR conjugates (SYN019 and SYN020) into the brain were almost ten-fold higher than that of BSA or Lf. These data show that conjugation of ADR to p97 does not affect the transport of p97 into the brain.

### Effect of p97-ADR Conjugate on Subcutaneous Tumors

**Figure 7:**
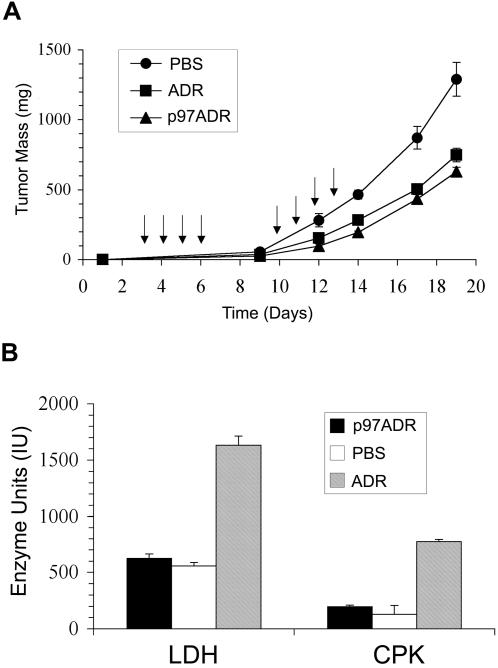
Mice bearing subcutaneous tumors were treated with p97-ADR conjugates. a) Tumor growth of subcutaneous C6 glioma mass in mice treated with p97-ADR conjugate SYN002 (4 mg/kg ADR, σ-σ), ADR (4 mg/kg, ν-ν) or PBS (λ-λ). Arrows indicate injection schedule (also refer to Trial 1 in Table 1). (n = 9 for each treatment group) b) Activity of LDH and CPK in serum from mice treated with the p97-ADR conjugate SYN002 (4 mg/kg ADR), ADR alone (4 mg/kg) or PBS. Values represent mean values+standard deviation (n = 3).

Treatment of C6 gliomas grown subcutaneously in mice was performed with both p97-ADR conjugate (SYN002) and free ADR. Both significantly (p<0.01* ANOVA) inhibited tumor growth by over 50% during a 20 day period when compared to PBS treatment (Figure 7a). The ADR doses (4 mg/kg, total) used in this trial were lower than those typically used in chemotherapy (10–15 mg/kg). The SYN002 conjugate was slightly more effective than treatment with an equivalent amount of pure ADR.

### Effect of p97-ADR Conjugation on Cardiotoxicity

There were significant differences in the serum levels of the two markers of cardiac damage, LDH and CPK after treatment of the animals (Figure 7b), as measured at the end of the trial (day 20). Treatment with ADR alone increased the level of both of these markers by over three-fold. Treatment with an equivalent amount of ADR conjugated to p97 (SYN002) had little effect on the level of these markers of cardiac cell damage, implying that conjugating ADR to p97 may reduce the cardiotoxic effects of ADR treatment. In all trials, the body weight of the mice did not appear to be affected by the treatments when compared to PBS controls.

### Efficacy of p97-ADR Conjugates on Intracranial Tumors

**Figure 8:**
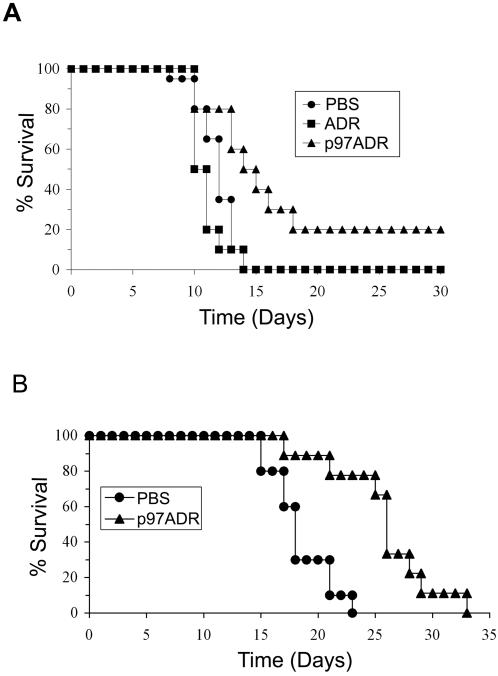
Mice bearing intracranial tumors were treated with p97-ADR conjugates. a) Percentage (%) survival of mice bearing intracranial ZR-75-1 mammary tumors and treated with p97-ADR conjugate SYN002 (5.5 mg/kg ADR, σ-σ), ADR (20 mg/kg, ν-ν) or PBS (λ-λ). b) Percentage (%) survival of mice bearing intracranial C6 glioma tumors and treated with p97-ADR conjugate SYN018 (0.49 mg/kg ADR, σ-σ) or PBS (λ-λ).

The dosing schedules and different trials are outlined in Table 2, while the results of the treatments are presented in Table 3. In Trial 2 (Figure 8a) where the mice with intracranial ZR-75-1 mammary tumors were treated with p97-ADR SYN002 via tail vein injections, mean and median survival times were increased by 77% and 20.8% compared to the PBS treated group. It is interesting that treatment with free ADR alone appeared to reduced the mean and median survival of the mice suggesting greater global organ and tissue toxicity due to free ADR in these mice. It should also be noted that the total ADR injected in the form of conjugate was four times less than that of free ADR. A higher dosage of p97-ADR might further increase survival rates. Two mice in the SYN002 treated group survived to 50 days and were reported, after autopsy, to be tumor-free survivors. In Trial 3 (Figure 8b) where mice with intracranial C6 gliomas were treated with SYN018 via tail vein injections, mean and median survival times were increased by 40% and 44% respectively compared to the PBS treated group. The efficacy of the p97-ADR conjugates, SYN002 and SYN018, did not appear to be affected by differences in the preparation and both were effective in the treatment of tumors. Significant increases in survival time were again achieved with relatively small total doses of ADR borne as p97-ADR. In Trial 4 where mice with intracranial C6 gliomas were treated with SYN002 via intrajugular vein injections, mean and median survival times were increased by 28% and 35% respectively when compared to the PBS treated group. In this trial, treatment with free ADR also improved the mean and median survival times of the mice but the improvement was considerably less than that of the p97-ADR (see Table 3), and resulted in higher over toxicity to the mice. Compared to Trial 2, efficacy of the p97-ADR did not appear to be affected by injection method, either tail vein or intrajugular vein. Therefore, the three studies showed that treatment with the p97-ADR conjugates were able to significantly extend the survival of mice with intracranial C6 gliomas or ZR-75-1 mammary tumors.

**Table 3:** p97-conjugated ADR increased survival times in mice with intracranial tumors.

Trial	Compound	Mean Survival In Days	% Change in Mean Survival	% Change in Median Survival	Log Rank Significance
**2 (Zr-75-1 Tumors)**	PBS	10	-	-	-
	ADR	9.24	−7.6	−12.5	p<0.05
	SYN002	17.7	77	20.8	p<0.005
**3 (C6 Tumors)**	PBS	20.2	-	-	-
	SYN018	28.3	40	44	p<0.001
**4 (C6 Tumors)**	PBS	21.8	-	-	-
	ADR	24.13	10.7	13.6	p<0.05
	SYN002	27.9	28	35	p<0.005

The percent increase in survival times for mice with free or conjugated ADR compared to controls treated with PBS clearly indicates that ADR conjugation to p97 results in a survival advantage for mice with intracranial tumors.

## Discussion

The present study describes the first protein based ‘ferrying’ system that can be used as a brain delivery vehicle for traversing therapeutically efficacious concentrations of drugs into the brain for the treatment of neurological disease.

We have demonstrated that recombinant human p97, injected into experimental mice, showed few signs of molecular breakdown in circulating blood, even after 8 hours. In time-course studies, we showed that 10 minutes after the injection of radio-iodinated p97, more radioactivity was detected in the brain than after injection of radio-labeled albumin control. One hour after injection, ^125^I-p97 radioactivity, normalized per mg of tissue wet weight, in the blood, kidney, bladder, liver, spleen, gallbladder, eye, heart and lung were all higher than in the central nervous system. The initial uptake of p97 in the kidney, liver and spleen increased rapidly within minutes of injection. However, after 1 hr, p97 was found to accumulate in the brain while its uptake in all other organs fell to the same level as the albumin control. Unlike the other organs, the brain continued to accumulate p97, even 24 hours post injection. Over this 24 hr period, the ratio of counts per minute per mg due to ^125^I-p97 in the brain compared to that in the plasma reached 10∶ 1. After 24 hr, the total accumulation of p97 in the brain reached 1–2% of the injected dose equivalent to the ratio of brain to body weight-the first carrier system to approximate this biological feat. This is a much higher accumulation than that others have observed for chemotherapeutics to date [15], [71], [72]. For example, less than 0.1% of morphine accumulates in the brain over time[73]. The organ distribution of ^125^I-p97, its brain accumulation over time and the fact that the BBB microvasculature lacks albumin receptors suggests that ^125^I-p97 entry into the brain is likely due to a receptor. Accumulation of homologous (murine) p97 would possibly be even greater than of the heterologous (human) p97 studied here and perhaps bodes well for transfer of this technology into a clinical application in humans. In the present study, the transport of p97 across the BBB without altering BBB permeability was confirmed using microscopy (Figure 5). The uptake of Alexa-, DIG- or gold-conjugated-p97 and unconjugated p97 by the brain was observed using confocal microscopy and EM (Figure 5). Consistent with studies using radio-labeled compounds [39], the BBB appeared to be more effective in limiting the entry of Tf into the brain than p97. The distribution of Tf was limited to microvessels, whereas p97 was observed in the cerebral parenchyma (Figure 5).

To determine whether p97 completely traverses the BBB or still resides within the circulation (mainly capillaries), the compartmental distribution of ^125^I-p97 after *i.v.* injection was analyzed by applying the capillary depletion method to the cerebral cortex. As the BBB isolates the cortex from all systemic influences, any systemically *i.v.* administered ^125^I-p97 recovered from the brain parenchyma must have penetrated the BBB from the circulation. Six hours after *i.v.* injection into mice, we found most ^125^I-p97 (over 80%) associated with the brain parenchyma rather than in the brain capillaries. The fluorescence distribution (Figure 5) makes it unlikely that p97 is attached to the luminal surface of the endothelial walls. These results indicate that ^125^I-p97 does not associate with the CNS vasculature but is transcytosed through the endothelial cells into the brain parenchyma, consistent with past findings [10], [44], [74].

Supporting the hypothesis that p97 enters the brain through a receptor-mediated process on the BBB, we have found that the rate of uptake into the brain of *i.v.* injected ^125^I-p97 can be inhibited in a dose related manner by an excess of unlabeled p97 [52]. This “cold block” signifies competition for a receptor, possibly a LRP receptor [52]. Using multiple-time regression analysis [57], [69] we found that the influx constant is higher for p97 (Figure 4) than that for albumin, which crosses the BBB through extracellular pathways [70], again suggesting the presence of a receptor mediated system of ^125^I-p97 transport, as suggested in previous reports [49], [74]. In contrast, the addition of unlabeled p97 did not change the entry rate of^ 99^Tc-albumin indicating that there was no disruption of the BBB due to injection of these radiolabeled proteins. We have also shown that PTAX-conjugated-p97 is transported to the brain (Figure 5f), whereas free PTAX could not be detected. We were able to identify PTAX and its metabolites in mouse brains after a cycle of 5 injections of p97-PTAX (Table 1).

Standard clinical chemotherapy for brain tumors includes highly lipophilic alkylating agents such as nitrosourea and temozolomide [72] which are able to cross the BBB. However, their effectiveness is limited by the low sensitivity of primary tumors to these drugs [75]. As a rigorous test of p97 as a drug delivery agent, we chose to use anti-cancer drugs with a high therapeutic index whose path to brain tissue from the circulation is normally blocked by the BBB. Thus we chose to study Paclitaxel (PTAX) and Doxorubicin (formerly Adriamycin (ADR)). PTAX was first identified in the bark of the Pacific yew tree, *Taxus brevifolia*, by Monroe E. Wall and Mansukh C. Wani [76]. Initially, there were concerns regarding potential environmental impact when it was learned that 1,200 kg of pacific yew tree bark yielded only 10 g of pure material [77]. In order to address this problem, Pierre Potier and then Robert A. Holton, completed the first semi-synthesis of PTAX from the needles of the English yew tree, *Taxus baccata* [77]. This was followed by the first total synthesis of PTAX by Holton and his colleagues [78], [79]. Subsequently, fermentation techniques were developed to allow large scale production without invoking wide-scale decimation of Pacific or European Yew populations [80]. PTAX blocks cell division by binding and stabilizing microtubules, which comprise the cytoskeleton and the mitotic spindle [81]. It is used in the treatment of lung, ovarian, breast, head and neck cancers and advanced forms of Kaposi's sarcoma [77]. Unfortunately, PTAX has no beneficial clinical effect in halting the growth of brain tumors or extending the life of patients with brain tumors largely due to its inability to traverse the BBB [82], [83]. We find that while p97-PTAX conjugates effectively cross the BBB, we chose to study the therapeutic effects of ADR because PTAX lacks tumorcidal or cytostatic activity against C6 gliomas [68].

Another common chemotherapeutic agent studied here, ADR, is an anthracycline glycoside. It was derived from a red-colored antibiotic produced from a strain of *Streptomyces peucetius* by a group at Farmitalia Research Laboratories working near the shores of the Adriatic Sea [84]. ADR is commonly used to treat leukemias, Hodgkin's lymphoma as well as solid tumors such as cancers of the breast, stomach, bladder, lung, ovaries, thyroid and soft tissue sarcomas, multiple myeloma, and others [85]. It has a number of possible mechanisms of action, including inhibition of DNA replication after it intercalates with DNA. ADR inhibits the progression of the enzyme topoisomerase II after it has broken the DNA chain to allow replication; it prevents the DNA double helix from being religated [86], [87]. ADR is 500–3000 times more effective against glioma cells *in vitro* [88] than *in vivo*, but the presence of efflux pumps localized at the blood–brain barrier renders it ineffective against tumors in the CNS [12], [89]. In addition, its effectiveness is limited by a short half-life *in vivo*, a large apparent volume of distribution that results in low brain tumor accumulation [90] and toxic side effects on normal organs, including the heart [91]. Therefore, in the chemotherapeutic trials conducted here, ADR conjugates were particularly good candidates for evaluation because free ADR is excluded from the brain by Pgp1 efflux activity and because ADR is completely ineffective in treating brain tumors [92] because of hindrance by the BBB. The p97-ADR conjugates were stable in serum (>18 hours) and two different batches had very similar properties. In all the trials, the total dose of conjugated ADR injected into mice was significantly lower than that of a typical therapeutic dose of free ADR (see Table 2). In Trial 1, we showed that p97-ADR (SYN002) was as effective in inhibiting subcutaneous glioma tumor growth as an equivalent amount of free ADR (over 50% reduction in tumor mass over a period of 19 days) (Figure 7a). The data further demonstrated that therapeutic amounts of ADR [30], could be delivered into the brain upon conjugation to p97. In Trial 5 (Table 2 and Figure 6b), we showed that similar amounts of p97 as p97-ADR conjugates (SYN019 and SYN020) were transported into the brains of mice after tail vein injection. Both were transported into the brain at significantly higher levels (approximately 10 fold higher) than lactoferrin control [48].

Free ADR has been shown to be cardiotoxic in some cases [64], [65] and it can cause neuro-toxicity when administered to the brain via partial permeabilisation of the BBB [93], limiting the amount of ADR tolerated by human patients. In Trial 1, two enzyme markers of cardiotoxicity, LDH and CPK, were monitored. When free ADR was administered, there was a significant increase in both markers. In contrast, when p97-ADR was administered, the levels of both markers were similar to that seen after PBS injections, indicating that conjugation to p97 could reduce the toxic effects of ADR in future clinical settings. It has been noted by others [33] that ADR cardiotoxicity can be reduced by conjugation of ADR to the peptides D-penetratin and SynB1. In addition, delivery of ADR via liposomes has resulted in reduced cardiotoxic effects [92]. It is not clear, whether conjugation or delivery by liposomes reduces the cardiotoxic effects of ADR by altering biodistribution or limiting the entry of ADR into cardiac tissue.

In the intracranial trials (Trials 2–4), treatment at 1 to 3 day intervals with the p97-ADR conjugates resulted in significant prolongation of survival of mice with brain tumors when compared to treatment with ADR alone. In Trial 2, mice bearing ZR-75-1 mammary tumor cells were treated, starting the next day, with the p97-ADR conjugate, SYN002. Untreated, the average mouse survival time was only approximately 10 days. Treatment with the conjugate raised mean and median survivals to 77% and 20% respectively. Notably, two mice out of the 10 in the treatment group survived for over 50 days and were tumor-free at autopsy, by careful gross (non-histological) inspection. In trials 3 and 4, mice were injected intracranially with rat C6 glioma cells and treated with the p97-ADR conjugates SYN002 and SYN018 either via tail vein injection (Trial 3) or via intra-jugular injections (Trial 4). These brain tumors grew slower than ZR-75-1 tumors resulting in an average survival time of 21 days for untreated animals [67], [94]. In both trials, significant increases in mean and median survival were achieved with p97-ADR (Table 3). In Trial 3, p97-ADR treatment allowed the mice to live more than 8 days longer than PBS control-treated animals, resulting in a 40% increase in mean survival and a 44% increase in median survival over PBS controls. In Trial 4, also found that p97-ADR treatment allowed the tumor-bearing mice to live 8 days longer than PBS controls and 4 days longer than mice treated with free ADR. This worked out to a 28% increase in mean survival (35% increase in medial survival) over PBS, and a 16% increase in mean survival over free ADR. In other studies (not shown), treatment with p97 alone had no effect on tumor suppression. The total ADR injected into mice over a given trial was significantly less than a typical human clinical therapeutic dose administered as a single bolus (20 mg/kg or 60 mg/m^2^ versus 23.2 mg/kg or 70 mg/m^2^) [95], [96]. Therefore, future trials will test whether higher total ADR dosage, delivered as p97 conjugates, will further increase survival times. Nevertheless, these studies demonstrate that p97, once conjugated to drug compounds, can cross the blood brain barrier at a similar rate to its normal (unconjugated) physiological efficiency and that the molecule remains intact following transcytosis but its payload is fully tumoricidally active once placed inside the brain and behind the BBB.

The presence of the BBB, although protective in design, not surprisingly limits the effectiveness of therapeutic drugs directed at the treatment of diseases of the brain. The BBB effectively prevents most drugs from reaching the brain, whether or not they are intravenously injected. Situations where this becomes most evident are during therapeutic applications such as treatment of neurophysiologic disorders (including lysosomal storage diseases), brain cancers (neuroblastomas, gliomas), infections and inflammation, trauma, and delivery of constructs used for gene therapy. Four main routes for molecules to enter the brain include the transcellular pathway (crossing through individual endothelial cells), the paracellular pathway (crossing between adjacent endothelial cells), intrathecal injection into the space surrounding the spinal cord or intracerebral injection (mechanical disruption of BBB by needle). Certain molecules may passively diffuse through a lipid membrane, employing the transcellular route. Increased lipid solubility, lack of polarizability, changes in hydrogen bonding ability and molecular size may make this route possible. Generally, the more lipid-soluble a molecule is, the more readily it moves from the aqueous environment of the blood across the nonpolar (lipid) environment of the endothelial cell membrane and enters the brain [97]. Counteracting influences that may slow diffusion include pH, temperature (unlikely to be of pharmacological importance in the mammalian brain) and retention in the blood due to protein binding [97]. Molar excess refraction, or the approximate measure of total volume, also appears plays a role in passive diffusion since diffusion coefficients are inversely related to molecular size, such that smaller compounds diffuse faster than larger compounds. Interestingly, permeability across the BBB appears to be inversely correlated to molecular size, where the addition of nonpolar groups to smaller molecules actually increases their ability to cross lipid barriers [97]. The disadvantage is that with this method, increased penetration across all membranes, not only the BBB, is increased non-specifically.

Another general route for transfer into tissues is the paracellular route, between capillary endothelial cells in this case. However, in the BBB, the paracellular route is blocked by tight junctions, preventing diffusion of even small molecules such as ions and water. Temporary deliberate disruption of this barrier to deliver drugs may be achieved by intravenous injections of hyperosmotic solutions (*i.e.* 2M mannitol) [98]–[100] or of biologically active agents such as bradykinin or angiotensins [97], [101]. However, the permeabilisation effects are neither confined to the brain, so there is no drug tissue-selectivity; nor are they molecularly selective within the brain. Ionic/chemical imbalances ensue, and plasma proteins can not find their way into the brain extracellular fluid. Osmotic shock is therefore bound to be harmful due to the non-specificity of the proteins and other molecules which may also enter the brain extracellular fluid along with the drug of choice. Highly active efflux systems also limit the passive diffusion of molecules across the BBB. Three families of large glycosylated membrane proteins that act at the BBB include Pgp1, multi-drug resistant proteins (MRP1 and MRP5), and organic anion transporting polypeptides (OAT3 and OATP1) [97], [102]. All exhibit broad substrate specificity. Some progress is being made in animal models to identify compounds that temporarily inhibit efflux pumps while momentarily allowing normally excluded therapeutic compounds to enter the brain[103], though the apparent draw back appears to be unforeseen dose related toxicity.

Currently, direct intra-cerebral delivery (infusion or implantation) is the only effective brain treatment to allow increased drug delivery [101], [104] that was previously described. However, the volume that can be delivered is limited (as compared to intravenous injection), and the drug must still diffuse to the brain parenchyma from the site of deposit to be effective. A high risk of infection and high neurosurgical costs are also involved in this approach. Other procedures for brain delivery, including delivery of microcapsulated drugs [105]–[107], and the exploitation of specific membrane transporters for conjugated drugs are being investigated. Furthermore, it has been found that antibodies conjugated to drugs, can cross the BBB as a result of their interaction with specific receptors, which suggests that such conjugates may be of value in the delivery of systemic-borne therapeutic agents to the brain [31], [107], [108]. Issues related to immune hypersensitivity using this approach must ultimately be addressed, perhaps by “humanizing” the potential antibody carrier, in order to use these conjugates repeatedly. Clearly, non-invasive methods based on using endogenous BBB shuttling compounds for the introduction of therapeutic compounds across the BBB and into brain parenchyma should be developed as therapeutic intervention in many neuropathologies may only then be achieved.

Melanotransferrin (p97) is one such endogenous shuttling protein that has clear potential as BBB drug delivery vehicle. It appears to offer many advantages over existing delivery molecules or systems. First, p97 is a protein found at low levels (<10 ng/mL) [54] in the blood of most normal individuals. Alzheimer's patients appear to be the lone exception identified to date, where levels in the blood may be two or more fold higher [44], [54], [109]. Therefore, inhibition of injected p97-drug conjugates by endogenous material that could competitively occupy receptors at the BBB appears to be minimal. Secondly, since the p97 appears to traverse the BBB as part of its normal function, its use in delivering drugs is not likely to result in p97-associated toxicity, though this has yet to be proven in a clinical setting. Thirdly, exogenously-introduced p97 can be expected to localize in concert with the tissue distribution of the target receptor. Thus it appears to localize to brain microvasculature and subsequently concentrates in brain parenchyma [52]. Therapies for neurological diseases can thereby be preferentially targeted to the brain. Fourthly, because it is an autologous human protein, repeated treatments are unlikely to result in immune hypersensitivity or in elimination by neutralizing antibodies in clinical therapies. Finally, the transport of p97 and p97-conjugates does not appear to be adversely affected by the antiport activities of pumps such as Pgp-1.

This study demonstrates the unique potential of using p97, as a ‘Trojan horse’ to ferry normally excluded therapeutic compounds, through the battlements of the BBB, thereby allowing forbidden cargo to traverse from the blood and emerge in the brain. The utilization of p97 as a shuttling platform is thus a new paradigm for carrier-mediated transport into the brain. New avenues should now be open to explore the generalized use of p97 to transport therapeutic compounds into the brain for the treatment of a variety of chronic and acute CNS diseases.

## References

[1] Neuwelt E, Abbott NJ, Abrey L, Banks WA, Blakley B (2008). Strategies to advance translational research into brain barriers.. Lancet Neurol.

[2] Brightman MW, Reese TS (1969). Junctions between intimately apposed cell membranes in the vertebrate brain.. J Cell Biol.

[3] Reese TS, Karnovsky MJ (1967). Fine structural localization of a blood-brain barrier to exogenous peroxidase.. J Cell Biol.

[4] Stewart PA (2000). Endothelial vesicles in the blood-brain barrier: are they related to permeability?. Cell Mol Neurobiol.

[5] Saunders NR, Knott GW, Dziegielewska KM (2000). Barriers in the immature brain.. Cell Mol Neurobiol.

[6] Saunders NR, Habgood MD, Dziegielewska KM (1999). Barrier mechanisms in the brain, II. Immature brain.. Clin Exp Pharmacol Physiol.

[7] Saunders NR, Habgood MD, Dziegielewska KM (1999). Barrier mechanisms in the brain, I. Adult brain.. Clin Exp Pharmacol Physiol.

[8] Pardridge WM, Boado RJ, Farrell CR (1990). Brain-type glucose transporter (GLUT-1) is selectively localized to the blood-brain barrier. Studies with quantitative western blotting and in situ hybridization.. J Biol Chem.

[9] Risau W, Hallmann R, Albrecht U (1986). Differentiation-dependent expression of proteins in brain endothelium during development of the blood-brain barrier.. Dev Biol.

[10] Rothenberger S, Food MR, Gabathuler R, Kennard ML, Yamada T (1996). Coincident expression and distribution of melanotransferrin and transferrin receptor in human brain capillary endothelium.. Brain Res.

[11] Raub TJ, Newton CR (1991). Recycling kinetics and transcytosis of transferrin in primary cultures of bovine brain microvessel endothelial cells.. J Cell Physiol.

[12] Tsuji A (1998). P-glycoprotein-mediated efflux transport of anticancer drugs at the blood-brain barrier.. Ther Drug Monit.

[13] Salcman M, Kaye A, Laws E (1995). Glioblastoma and malignant astrocytoma;.

[14] Ambruosi A, Gelperina S, Khalansky A, Tanski S, Theisen A (2006). Influence of surfactants, polymer and doxorubicin loading on the anti-tumour effect of poly(butyl cyanoacrylate) nanoparticles in a rat glioma model.. J Microencapsul.

[15] Smith MW, Gumbleton M (2006). Endocytosis at the blood-brain barrier: from basic understanding to drug delivery strategies.. J Drug Target.

[16] Michaelis K, Hoffmann MM, Dreis S, Herbert E, Alyautdin RN (2006). Covalent linkage of apolipoprotein e to albumin nanoparticles strongly enhances drug transport into the brain.. J Pharmacol Exp Ther.

[17] Gao K, Jiang X (2006). Influence of particle size on transport of methotrexate across blood brain barrier by polysorbate 80-coated polybutylcyanoacrylate nanoparticles.. Int J Pharm.

[18] Chen Y, Dalwadi G, Benson HA (2004). Drug delivery across the blood-brain barrier.. Curr Drug Deliv.

[19] Abulrob A, Sprong H, Van Bergen en Henegouwen P, Stanimirovic D (2005). The blood-brain barrier transmigrating single domain antibody: mechanisms of transport and antigenic epitopes in human brain endothelial cells.. J Neurochem.

[20] Roney C, Kulkarni P, Arora V, Antich P, Bonte F (2005). Targeted nanoparticles for drug delivery through the blood-brain barrier for Alzheimer's disease.. J Control Release.

[21] Olivier JC (2005). Drug transport to brain with targeted nanoparticles.. NeuroRx.

[22] Tsuji A (2005). Small molecular drug transfer across the blood-brain barrier via carrier-mediated transport systems.. NeuroRx.

[23] Egleton RD, Davis TP (2005). Development of neuropeptide drugs that cross the blood-brain barrier.. NeuroRx.

[24] Kas HS (2004). Drug delivery to brain by microparticulate systems.. Adv Exp Med Biol.

[25] Kreuter J (2004). Influence of the surface properties on nanoparticle-mediated transport of drugs to the brain.. J Nanosci Nanotechnol.

[26] Bartus R (1999). The blood-brain barrier as a target for pharmacological modulation.. Curr Opin Drug Discov Dev.

[27] McAllister LD, Doolittle ND, Guastadisegni PE, Kraemer DF, Lacy CA (2000). Cognitive outcomes and long-term follow-up results after enhanced chemotherapy delivery for primary central nervous system lymphoma.. Neurosurgery.

[28] Kinoshita M, McDannold N, Jolesz FA, Hynynen K (2006). Noninvasive localized delivery of Herceptin to the mouse brain by MRI-guided focused ultrasound-induced blood-brain barrier disruption.. Proc Natl Acad Sci U S A.

[29] Ohkawa K, Hatano T, Yamada K, Joh K, Takada K (1993). Bovine serum albumin-doxorubicin conjugate overcomes multidrug resistance in a rat hepatoma.. Cancer Res.

[30] Ohnishi T, Tamai I, Sakanaka K, Sakata A, Yamashima T (1995). In vivo and in vitro evidence for ATP-dependency of P-glycoprotein-mediated efflux of doxorubicin at the blood-brain barrier.. Biochem Pharmacol.

[31] Jefferies WA, Brandon MR, Hunt SV, Williams AF, Gatter KC (1984). Transferrin receptor on endothelium of brain capillaries.. Nature.

[32] Pardridge WM (1998). CNS drug design based on principles of blood-brain barrier transport.. J Neurochem.

[33] Rousselle C, Clair P, Lefauconnier JM, Kaczorek M, Scherrmann JM (2000). New advances in the transport of doxorubicin through the blood-brain barrier by a peptide vector-mediated strategy.. Mol Pharmacol.

[34] Coloma MJ, Lee HJ, Kurihara A, Landaw EM, Boado RJ (2000). Transport across the primate blood-brain barrier of a genetically engineered chimeric monoclonal antibody to the human insulin receptor.. Pharm Res.

[35] Lee HJ, Engelhardt B, Lesley J, Bickel U, Pardridge WM (2000). Targeting rat anti-mouse transferrin receptor monoclonal antibodies through blood-brain barrier in mouse.. J Pharmacol Exp Ther.

[36] Trail PA, Willner D, Knipe J, Henderson AJ, Lasch SJ (1997). Effect of linker variation on the stability, potency, and efficacy of carcinoma-reactive BR64-doxorubicin immunoconjugates.. Cancer Res.

[37] Kratz F, Beyer U, Roth T, Tarasova N, Collery P (1998). Transferrin conjugates of doxorubicin: synthesis, characterization, cellular uptake, and in vitro efficacy.. J Pharm Sci.

[38] Pardridge WM, Buciak JL, Friden PM (1991). Selective transport of an anti-transferrin receptor antibody through the blood-brain barrier in vivo.. J Pharmacol Exp Ther.

[39] Friden PM, Walus LR (1993). Transport of proteins across the blood-brain barrier via the transferrin receptor.. Adv Exp Med Biol.

[40] Moos T, Morgan EH (2001). Restricted transport of anti-transferrin receptor antibody (OX26) through the blood-brain barrier in the rat.. J Neurochem.

[41] Jefferies WA, Brandon MR, Williams AF, Hunt SV (1985). Analysis of lymphopoietic stem cells with a monoclonal antibody to the rat transferrin receptor.. Immunology.

[42] Brown JP, Hewick RM, Hellstrom I, Hellstrom KE, Doolittle RF (1982). Human melanoma-associated antigen p97 is structurally and functionally related to transferrin.. Nature.

[43] Food MR, Rothenberger S, Gabathuler R, Haidl ID, Reid G (1994). Transport and expression in human melanomas of a transferrin-like glycosylphosphatidylinositol-anchored protein.. J Biol Chem.

[44] Yamada T, Tsujioka Y, Taguchi J, Takahashi M, Tsuboi Y (1999). Melanotransferrin is produced by senile plaque-associated reactive microglia in Alzheimer's disease.. Brain Res.

[45] Pardridge WM (1998). Introduction to the blood-brain barrier: methodology, biology, and pathology..

[46] Lee HJ, Zhang Y, Zhu C, Duff K, Pardridge WM (2002). Imaging brain amyloid of Alzheimer disease in vivo in transgenic mice with an Abeta peptide radiopharmaceutical.. J Cereb Blood Flow Metab.

[47] Pardridge WM (1991). Advances in cell biology of blood-brain barrier transport.. Semin Cell Biol.

[48] Fillebeen C, Descamps L, Dehouck MP, Fenart L, Benaissa M (1999). Receptor-mediated transcytosis of lactoferrin through the blood-brain barrier.. J Biol Chem.

[49] Demeule M, Poirier J, Jodoin J, Bertrand Y, Desrosiers RR (2002). High transcytosis of melanotransferrin (P97) across the blood-brain barrier.. J Neurochem.

[50] Pardridge WM, Eisenberg J, Yang J (1987). Human blood-brain barrier transferrin receptor.. Metabolism.

[51] Demeule M, Bertrand Y, Michaud-Levesque J, Jodoin J, Rolland Y (2003). Regulation of plasminogen activation: a role for melanotransferrin (p97) in cell migration.. Blood.

[52] Moroo I, Ujiie M, Walker BL, Tiong JW, Vitalis TZ (2003). Identification of a novel route of iron transcytosis across the mammalian blood-brain barrier.. Microcirculation.

[53] Yang J, Tiong J, Kennard M, Jefferies WA (2004). Deletion of the GPI pre-anchor sequence in human p97–a general approach for generating the soluble form of GPI-linked proteins.. Protein Expr Purif.

[54] Kennard ML, Feldman H, Yamada T, Jefferies WA (1996). Serum levels of the iron binding protein p97 are elevated in Alzheimer's disease.. Nat Med.

[55] Hellstrom I, Brown JP, Hellstrom KE (1983). Melanoma-associated antigen p97 continues to be expressed after prolonged exposure of cells to specific antibody.. Int J Cancer.

[56] Blasberg RG, Patlak CS, Fenstermacher JD (1983). Selection of experimental conditions for the accurate determination of blood–brain transfer constants from single-time experiments: a theoretical analysis.. J Cereb Blood Flow Metab.

[57] Patlak CS, Blasberg RG, Fenstermacher JD (1983). Graphical evaluation of blood-to-brain transfer constants from multiple-time uptake data.. J Cereb Blood Flow Metab.

[58] Pan W, Vallance K, Kastin AJ (1999). TGFalpha and the blood-brain barrier: accumulation in cerebral vasculature.. Exp Neurol.

[59] Gutierrez EG, Banks WA, Kastin AJ (1993). Murine tumor necrosis factor alpha is transported from blood to brain in the mouse.. J Neuroimmunol.

[60] Bicamumpaka C, Page M (1998). In vitro cytotoxicity of paclitaxel-transferrin conjugate on H69 cells.. Oncol Rep.

[61] Sparreboom A, van Tellingen O, Nooijen WJ, Beijnen JH (1995). Determination of paclitaxel and metabolites in mouse plasma, tissues, urine and faeces by semi-automated reversed-phase high-performance liquid chromatography.. J Chromatogr B Biomed Appl.

[62] Royer I, Alvinerie P, Armand JP, Ho LK, Wright M (1995). Paclitaxel metabolites in human plasma and urine: identification of 6 alpha-hydroxytaxol, 7-epitaxol and taxol hydrolysis products using liquid chromatography/atmospheric-pressure chemical ionization mass spectrometry.. Rapid Commun Mass Spectrom.

[63] Kennard ML, Food MR, Jefferies WA, Piret JM (1993). Controlled release process to recover heterologous glycosylphosphatidylinositol membrane anchored proteins from CHO cells.. Biotechnology and Bioengineering.

[64] Shan K, Lincoff AM, Young JB (1996). Anthracycline-induced cardiotoxicity.. Ann Intern Med.

[65] Papoian T, Lewis W (1990). Adriamycin cardiotoxicity in vivo. Selective alterations in rat cardiac mRNAs.. Am J Pathol.

[66] Saleh M, Davis ID, Wilks AF (1997). The paracrine role of tumour-derived mIL-4 on tumour-associated endothelium.. Int J Cancer.

[67] Kaye AH, Morstyn G, Gardner I, Pyke K (1986). Development of a xenograft glioma model in mouse brain.. Cancer Res.

[68] Silbergeld DL, Chicoine MR, Madsen CL (1995). In vitro assessment of Taxol for human glioblastoma: chemosensitivity and cellular locomotion.. Anticancer Drugs.

[69] Blasberg RG, Kobayashi T, Horowitz M, Rice JM, Groothuis D (1983). Regional blood-to-tissue transport in ethylnitrosourea-induced brain tumors.. Ann Neurol.

[70] Banks WA, Broadwell RD (1994). Blood to brain and brain to blood passage of native horseradish peroxidase, wheat germ agglutinin, and albumin: pharmacokinetic and morphological assessments.. J Neurochem.

[71] Chertok B, Moffat BA, David AE, Yu F, Bergemann C (2008). Iron oxide nanoparticles as a drug delivery vehicle for MRI monitored magnetic targeting of brain tumors.. Biomaterials.

[72] Zhou Q, Guo P, Kruh GD, Vicini P, Wang X (2007). Predicting human tumor drug concentrations from a preclinical pharmacokinetic model of temozolomide brain disposition.. Clin Cancer Res.

[73] Strandberg JJ, Kugelberg FC, Alkass K, Gustavsson A, Zahlsen K (2006). Toxicological analysis in rats subjected to heroin and morphine overdose.. Toxicol Lett.

[74] Moroo I, Ujiie M, Walker BL, Tiong JWC, Vitalis TZ (2003). Identification of a novel route of iron transcytosis across the mammalian blood-brain barrier.. Microcirculation.

[75] Zhou Q, Guo P, Wang X, Nuthalapati S, Gallo JM (2007). Preclinical pharmacokinetic and pharmacodynamic evaluation of metronomic and conventional temozolomide dosing regimens.. J Pharmacol Exp Ther.

[76] Wall ME, Wani MC (1995). Camptothecin and taxol: discovery to clinic–thirteenth Bruce F. Cain Memorial Award Lecture.. Cancer Res.

[77] Goodman J, Walsh V (2001). The story of taxol : nature and politics in the pursuit of an anti-cancer drug..

[78] Holton RA, Kim H-B, Somoza C, Liang F, Biediger RJ (1994). First total synthesis of taxol. 2. Completion of the C and D rings.. Journal of American Chemical Society.

[79] Holton RA, Somoza C, Kim H-B, Liang F, Biediger RJ (1994). First Total Synthesis of Taxol. 1. Functionalization of the B Ring.. Journal of American Chemical Society.

[80] Bristol-MyersSquibbCompany (2004). http://www.epa.gov/greenchemistry/pubs/pgcc/winners/gspa04.html.

[81] Holton RA, Biediger RJ, Boatman PD, Suffness M (1995). Semisynthesis of Taxol and Taxotere;.

[82] Heimans JJ, Vermorken JB, Wolbers JG, Eeltink CM, Meijer OW (1994). Paclitaxel (Taxol) concentrations in brain tumor tissue.. Ann Oncol.

[83] Fine RL, Chen J, Balmaceda C, Bruce JN, Huang M (2006). Randomized study of paclitaxel and tamoxifen deposition into human brain tumors: implications for the treatment of metastatic brain tumors.. Clin Cancer Res.

[84] Arcamone F, Cassinelli G, Fantini G, Grein A, Orezzi P (1969). Adriamycin, 14-hydroxydaunomycin, a new antitumor antibiotic from S. peucetius var. caesius.. Biotechnol Bioeng.

[85] MayoClinic(Micromedex) (2007). http://www.mayoclinic.com/health/drug-information/DR600581.

[86] Fornari FA, Randolph JK, Yalowich JC, Ritke MK, Gewirtz DA (1994). Interference by doxorubicin with DNA unwinding in MCF-7 breast tumor cells.. Mol Pharmacol.

[87] Momparler RL, Karon M, Siegel SE, Avila F (1976). Effect of adriamycin on DNA, RNA, and protein synthesis in cell-free systems and intact cells.. Cancer Res.

[88] Wolff JE, Trilling T, Molenkamp G, Egeler RM, Jurgens H (1999). Chemosensitivity of glioma cells in vitro: a meta analysis.. J Cancer Res Clin Oncol.

[89] Takamiya Y, Abe Y, Tanaka Y, Tsugu A, Kazuno M (1997). Murine P-glycoprotein on stromal vessels mediates multidrug resistance in intracerebral human glioma xenografts.. Br J Cancer.

[90] Speth PA, van Hoesel QG, Haanen C (1988). Clinical pharmacokinetics of doxorubicin.. Clin Pharmacokinet.

[91] Brigger I, Morizet J, Laudani L, Aubert G, Appel M (2004). Negative preclinical results with stealth nanospheres-encapsulated Doxorubicin in an orthotopic murine brain tumor model.. J Control Release.

[92] Sharma US, Sharma A, Chau RI, Straubinger RM (1997). Liposome-mediated therapy of intracranial brain tumors in a rat model.. Pharm Res.

[93] Kondo A, Inoue T, Nagara H, Tateishi J, Fukui M (1987). Neurotoxicity of adriamycin passed through the transiently disrupted blood-brain barrier by mannitol in the rat brain.. Brain Res.

[94] Saleh M, Wiegmans A, Malone Q, Stylli SS, Kaye AH (1999). Effect of in situ retroviral interleukin-4 transfer on established intracranial tumors.. J Natl Cancer Inst.

[95] OncologyTools http://www.fda.gov/cder/cancer/animalframe.htm

[96] MedicineOnline http://www.meds.com/leukemia/idamycin/adriamycin.html

[97] Habgood MD, Begley DJ, Abbott NJ (2000). Determinants of passive drug entry into the central nervous system.. Cell Mol Neurobiol.

[98] Neuwelt EA, Maravilla KR, Frenkel EP, Rapaport SI, Hill SA (1979). Osmotic blood-brain barrier disruption. Computerized tomographic monitoring of chemotherapeutic agent delivery.. J Clin Invest.

[99] Neuwelt EA, Frenkel EP, Diehl J, Vu LH, Rapoport S (1980). Reversible osmotic blood-brain barrier disruption in humans: implications for the chemotherapy of malignant brain tumors.. Neurosurgery.

[100] Neuwelt EA, Maravilla KR, Frenkel EP, Barnett P, Hill S (1980). Use of enhanced computerized tomography to evaluate osmotic blood-brain barrier disruption.. Neurosurgery.

[101] Temsamani J, Rousselle C, Rees AR, Scherrmann JM (2001). Vector-mediated drug delivery to the brain.. Expert Opin Biol Ther.

[102] Ayrton A, Morgan P (2001). Role of transport proteins in drug absorption, distribution and excretion.. Xenobiotica.

[103] Hubensack M, Muller C, Hocherl P, Fellner S, Spruss T (2008). Effect of the ABCB1 modulators elacridar and tariquidar on the distribution of paclitaxel in nude mice.. J Cancer Res Clin Oncol.

[104] Cressant A, Desmaris N, Verot L, Brejot T, Froissart R (2004). Improved behavior and neuropathology in the mouse model of Sanfilippo type IIIB disease after adeno-associated virus-mediated gene transfer in the striatum.. J Neurosci.

[105] Rapoport SI (2000). Osmotic opening of the blood-brain barrier: principles, mechanism, and therapeutic applications.. Cell Mol Neurobiol.

[106] Pardridge WM (2007). Blood-brain barrier delivery of protein and non-viral gene therapeutics with molecular Trojan horses.. J Control Release.

[107] Boado RJ, Zhang Y, Zhang Y, Xia CF, Pardridge WM (2007). Fusion antibody for Alzheimer's disease with bidirectional transport across the blood-brain barrier and abeta fibril disaggregation.. Bioconjug Chem.

[108] Ueda F, Raja KB, Simpson RJ, Trowbridge IS, Bradbury MW (1993). Rate of 59Fe uptake into brain and cerebrospinal fluid and the influence thereon of antibodies against the transferrin receptor.. J Neurochem.

[109] Kim DK, Seo MY, Lim SW, Kim S, Kim JW (2001). Serum melanotransferrin, p97 as a biochemical marker of Alzheimer's disease.. Neuropsychopharmacology.

