# Correction: A Unique Carrier for Delivery of Therapeutic Compounds beyond the Blood-Brain Barrier

**DOI:** 10.1371/annotation/8183042c-af40-4227-8a00-8f362de22220

**Published:** 2008-07-17

**Authors:** Delara Karkan, Cheryl Pfeifer, Timothy Z. Vitalis, Gavin Arthur, Maki Ujiie, Qingqi Chen, Sam Tsai, Gerrasimo Koliatis, Reinhard Gabathuler, Wilfred A. Jefferies

The symbols in the first and second sentences of the legend to Figure 8 are incorrect. The correct Figure 8 legend should read: a) Percentage (%) survival of mice bearing intracranial ZR-75-1 mammary tumors and treated with p97-ADR conjugate SYN002 (5.5 mg/kg ADR, ▲-▲), ADR (20mg/kg, ■-■) or PBS (●-●). b) Percentage (%) survival of mice bearing intracranial C6 glioma tumors and treated with p97-ADR conjugate SYN018 (0.49mg/kg ADR, ▲-▲) or PBS (●-●). 

