# Correction: A Unique Carrier for Delivery of Therapeutic Compounds beyond the Blood-Brain Barrier

**DOI:** 10.1371/annotation/a08f47c0-6236-4116-821f-fa68c6b9a182

**Published:** 2008-07-17

**Authors:** Delara Karkan, Cheryl Pfeifer, Timothy Z. Vitalis, Gavin Arthur, Maki Ujiie, Qingqi Chen, Sam Tsai, Gerrasimo Koliatis, Reinhard Gabathuler, Wilfred A. Jefferies

The symbols in the first sentence of the Figure 7 legend are incorrect. The correct sentence is: a) Tumor growth of subcutaneous C6 glioma mass in mice treated with p97-ADR conjugate SYN002 (4mg/kg ADR, ▲-▲), ADR (4mg/kg, ■-■) or PBS (●-●). 

