# Correction: A Unique Carrier for Delivery of Therapeutic Compounds beyond the Blood-Brain Barrier

**DOI:** 10.1371/annotation/40c256ca-5d98-4ca9-a388-c9dac8f20924

**Published:** 2008-08-18

**Authors:** Delara Karkan, Cheryl Pfeifer, Timothy Z. Vitalis, Gavin Arthur, Maki Ujiie, Qingqi Chen, Sam Tsai, Gerrasimo Koliatis, Reinhard Gabathuler, Wilfred A. Jefferies

The competing interests section was incomplete. The following sentence should be included: RG wishes to disclose a competing interest in AngioChem Inc., subsequent to his involvement in the present study as Director of BBB Research at Synapse Technologies Inc.

